# Using HG1222 — A perspective into the ethics of collecting biospecimens

**DOI:** 10.1172/JCI173348

**Published:** 2023-07-17

**Authors:** Ayush Kumar

**Affiliations:** University of Massachusetts Chan Medical School, Worcester, Massachusetts, USA.

“The tumor bank has a new breast cancer sample, HG1222. Would you like to use it for your project?” Chris asked me as I walked into the lab. Normally, I would graciously accept the offer without hesitation, but today a tumor sample embodied more than just letters and symbols that I use for research.

Earlier that morning, I attended my preceptorship in the surgical oncology clinic, and Sally was the first patient I had a chance to talk to before the attending arrived. In her initial meeting a few weeks ago, she was distraught by her breast cancer diagnosis and hastily consented to have her tumor used for research after surgical removal. In her own words, the tumor was, “a parasite that I wanted removed,” and she was, “disinterested in its use.” However, after taking time to reorient her mental state, she felt uncertain about her initial decision and wanted more clarification. As an MD/PhD student, who does a lot of translational research, I instinctively wanted to share my positive experiences of using biospecimens in the pursuit of making new therapeutics for patients with cancer. However, I first decided to listen to her concerns and understand her perspective on donating tissues for research. Sally started by expressing her eagerness to help expand cancer research, but she had read about previous instances in which individuals donated his/her biospecimen and it negatively impacted the person and their family: the commercialization of HeLa cells initiated without the knowledge of Henrietta Lacks’ family; the sharing of specimens and genetic data throughout the world but not with the patient; and the rise of consumer DNA tests that allow users to publicize the data, which affects the privacy of the relatives of the customer.

With these examples in mind, the central theme of her concern was the juxtaposition of ideas of autonomy and anonymity, as involved with donating biospecimens. The current infrastructure in research focuses on protecting privacy of the patients while conceding a donor’s choice on the type of data collected and analyzed from the tissue sample. I had never concretely identified this issue faced by patients; it made me realize that the genomic analysis of donated tissue can reveal personal data, which is alarming to many people. She desperately inquired, “What do you think I should do?” At this point, I was at a crossroads in my response — either I advocate for donating her tumor and inadvertently downplay her concerns, or I suggest withdrawing her consent form and propagate mistrust in medical research.

As a physician-scientist in training, my career is focused on taking research from the “bench to bedside” to promote evidence-based medicine, which relies on data collected from patients’ samples. At an academic institution, I want to help my fellow researchers accomplish their goals by providing the necessary resources available in the clinic.

However, I also must uphold the “provider-patient” relationship and share with my patients the potential risks and limitations associated with donating biospecimens for research. Despite the dichotomy, Sally and I were able to have a candid conversation, discussing the benefits and risks associated with donating her tumor tissue. I stressed how important it is to make a decision that would make her the most comfortable and that I would be available to answer questions she had during that process. We agreed that the inappropriate use of patient samples has been well chronicled, but I further supported that the medical community has learned from these situations and focuses on preventing them from happening again. Specifically, I connected her with a representative from the tumor bank to answer some of her questions about the regulated usage of biospecimens at our institution. Our conversation eventually helped ease the tension associated with her indecisiveness. A few days later, Sally went forward with donating her tumor. This opportunity opened my eyes to the duality of being a physician-scientist: the “scientist” may focus on acquiring as many clinical samples as possible, whereas the “physician” needs to help the patient make an informed decision and never coerce him/her to donate tissue samples.

HG1222 can help answer another question about cancer biology, but I am oblivious of the patient’s personal beliefs about the genomic data that I will collect to answer it.

Patients provide “broad consent” to tumor banks, which need to protect patient privacy and allow researchers to collect data as they see fit. Hopefully, in the future, tumor banks can integrate secure information technology strategies that facilitate research-specific consent forms for patients about using their samples. This method would alleviate the concerns for patients about loss of autonomy and allow researchers to feel more comfortable using the samples for their projects.

## Version 1 07/17/2023

Electronic publication

